# LINE1 and its host: one cell’s junk is another cell’s treasure

**DOI:** 10.1038/s44318-024-00175-5

**Published:** 2024-07-29

**Authors:** John C Martinez, Andrei Seluanov, Vera Gorbunova

**Affiliations:** 1 Department of Biology, University of Rochester, Rochester, NY USA; 2 Department of Medicine, University of Rochester Medical Center, Rochester, NY USA

**Keywords:** Molecular Biology of Disease, Signal Transduction

## Abstract

New findings show that expression of the LINE1 retrotransposon RNA stimulates bone repair in mice and humans.

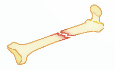

## Teaser

LINE1 (L1) retrotransposons have gained notoriety for their involvement in a slew of pathologies. While improper de-repression of L1 transcription may drive disease, mammalian cells have coopted L1 activation into mechanisms essential for cell function. New findings published by Mangiavacci et al (2024) in The EMBO Journal reveal the role of L1 RNA in stimulating osteogenic activity during bone repair in both mice and humans, highlighting a previously undescribed beneficial regulatory role for L1 activity.

**Figure 1:**
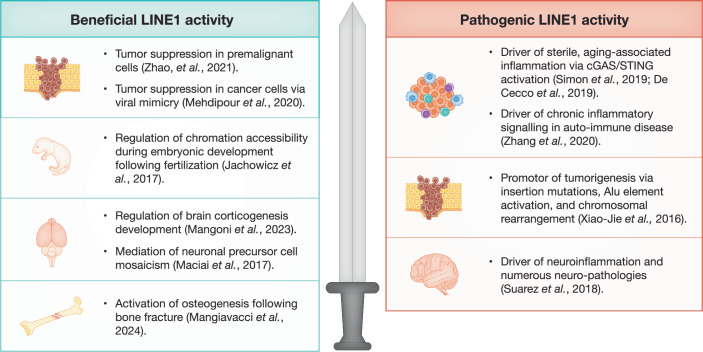
LINE1 activity, expression, and inflammation: a double-edged sword. L1 expression can lead to beneficial (left) and pathogenic (right) effects on cellular homeostasis, development and pathologic states. Beneficial effects include tumor suppression in premalignant cells and in cancer cells via viral mimicry; regulation of chromatin accessibility during embryonic development after fertilization; regulation of brain corticogenesis; and activation of osteogenesis following bone fracture as demonstrated in the study by Mangiavacci et al (2024) (Mangoni et al, 2023; Jachowicz et al, 2017). On the other hand, pathogenic effects of LINE1 include the promotion of tumorigenesis; induction of inflammation during aging and upon autoimmune disease; and induction of neuroinflammation and neuropathologies (Suarez et al, 2018).

L1 retrotransposons appear to have two personalities: that of a virus-like element capable of inducing disease, and an essential component that our cells have evolved to regulate numerous cellular processes. A substantial portion of recent research on L1 activity has focused on the pathological consequences of L1 de-repression. It is, however, becoming evident that L1 expression is an important mediator of beneficial cell processes (Fig. 1). A prime example of beneficial L1 expression is its role in tumor suppression. The blind mole rat, a long-lived rodent (maximum lifespan >21 years), expresses retrotransposable elements upon cellular hyperplasia, thus generating the formation of cytosolic DNA–RNA hybrids capable of simulating the cGAS/STING pathway and inducing cell death (Zhao et al, 2021; Mehdipour et al, 2020). Conversely, the interest in L1 expression within the context of disease is not without warrant. Aberrant de-repression of L1 elements has been correlated with autoimmune disorders, aging-associated inflammation, and cancer (Zhang et al, 2020; Simon et al, 2019; De Cecco et al, 2019) While the understanding of L1 biology and life cycle and processes have progressed significantly in the past decade, the impact of L1 on the host is far from being fully understood.

As the overall global population continues to age at an accelerated rate, aging-associated pathologies are poised to inflict an increasing burden on healthcare systems and patient’s overall quality of life. Osteoporosis, characterized by the progressive loss of bone mineral density and bone mass that results in increased frailty and susceptibility to fractures, already affects 200 million patients worldwide and is a leading cause of debilitation with age (Barnsley et al, 2021). There is an unmet need in pharmacological interventions promoting bone regeneration to reduce fracture susceptibility. A major hurdle in developing safe, effective treatments for osteoporosis stems from an abridged understanding of the underlying cellular mechanisms that regulate bone regeneration. Striking new findings published by Mangiavacci et al (2024) show that L1 RNA transcription is activated during fracture and plays a crucial role in stimulating bone mineralization in osteoclasts derived from both mice and humans. Perhaps the most captivating result from this work is that delivery of L1 RNA to osteoblasts results in a significant induction of bone mineralization compared to cells treated with a sham RNA. Importantly, this matrix formation is stimulated by L1 RNA, rather than L1 cDNA or DNA–RNA hybrids, which induces protein kinase R (PKR) phosphorylation of EIF2α. Thus, this mechanism is regulated upstream of cytosolic nucleic acid sensing pathways implicated in autoimmune disorders and aging-associated inflammation. While future work to determine the role and transcription levels of L1 RNA activity in osteoblasts specifically during the aging process is needed to determine if L1 RNA delivery is a potential regeneration therapy in vivo, these findings demonstrate significant advances in the understanding of osteoblast transposon activity. Further, the importance of L1 RNA following bone fracture highlights a previously undescribed beneficial regulatory role for L1 expression.

The authors begin by demonstrating via transcriptome analysis that retrotransposons become active following fracture, and specifically identify and organize the subfamilies based on their activity levels before, immediately after, and at various later timepoints during the bone healing process following injury in mice. Remarkably, L1 elements and long terminal repeats (LTRs) were inactive prior to fracture and became upregulated immediately after. Intrigued by their in vivo murine data, Mangiavacci et al next investigated if there were differences in retrotransposon activity from human donor bone marrow with patients categorized as healthy, osteopenic, or osteoporotic. These patients were also designated as containing high bone mineral density (BMD) or low BMD. Initial findings demonstrated that donors experiencing osteopenic or osteoporotic disease had decreased transposon expression relative to healthy donors. Further, donors separated as having high or low BMD experienced dramatically different levels of transposon in the bone marrow, with high BMD donors exhibiting increased TE activity. While other TE family expression levels, including SINEs, remained unchanged between donors, 30% of L1 and LTR subfamilies were found to be positively correlated with healthy BMD. These results strongly suggest L1s play a role in bone marrow stress response.

The distinct differences between healthy and unhealthy bone marrow L1 expression prompted the authors to investigate if L1 RNA was directly stimulating bone mineralization in osteoblasts. Utilizing osteoblasts differentiated in vitro from healthy donors, the authors transfected exogenous L1 RNA into cells. Remarkably, osteoblasts treated with L1 RNA, but not control RNA, induced mineralized matrix production in a dose-dependent manner. Importantly, using a Cy5-conjugated RNA, researchers visualized L1 RNAs eventually being secreted from the cell rather than forming cDNAs. Next, analyzing the effect of L1 RNA delivery to osteoblasts differentiated from osteoporotic patients, investigators once again observed matrix formation triggered in cells, regardless of their otherwise compromised anabolic activity. These results convey an exciting potential in harnessing L1 RNA to alleviate osteoporotic pathology.

Diving deeper into their findings, the authors then assessed the transcriptomic profile of osteoblasts treated with L1 or control RNA. Astoundingly, cells treated with L1 RNA mimicked GO terms typically enriched in the bone after fracture in vivo or differentiating osteoblasts in vitro. Demonstrating the similarity in pathways activated upon fracture and L1 RNA treatment of osteoblasts strengthens the hypothesis L1 activation plays a role in osteogenic repair. Next, the authors substantiate this pathway is specifically dependent on L1 RNA rather than L1 cDNA/hybrids or the cGAS/STING pathway by utilizing NRTIs to inhibit ORF2p reverse-transcriptase (RT) activity or a cGAS inhibitor. Neither treatment inhibited bone mineralization upon L1 RNA delivery. Given the link between aberrant cGAS signaling and degenerative inflammation, this is a crucial control that may also help explain the specificity of the osteogenic response to fracture rather than a more generalized cGAS/STING response (Gulen et al, 2023). Finally, the authors demonstrate that PKR, responsible in part for sensing double-stranded RNA generated by retrotransposons, binds to L1 RNA and inducing the phosphorylation of eIF2α to attenuate global protein synthesis in the cell (Boye and Grallert, 2020). After using mass spectrometry (MS) to demonstrate substantial protein reduction in L1 RNA treated osteoblasts, the authors used the PKR inhibitor C16 to successfully demonstrate inhibition of L1 RNA facilitated inflammatory gene activation and mineral matrix deposition. Thus, L1 RNA stimulates PKR phosphorylation of eIF2α to induce bone mineralization in osteoblasts.

Collectively, the findings presented by Mangiavacci et al (2024) yield fascinating insights into the role L1 activation plays in bone matrix formation. Upon fracture, de-repression of retrotransposon RNA, primarily L1s, in osteoblasts stimulates bone matrix formation by PKR activation and inhibition of protein synthesis. Importantly, L1 RNAs lead to activation of immune response genes associated with bone fracture repair independent of ORF2p reverse-transcriptase activity or cGAS/STING. Successful matrix formation in osteoblasts differentiated from both healthy and osteoporotic donor bone marrow upon L1 RNA treatment represents an intriguing potential therapy for osteoporosis. Ultimately, these results emphasize a previously undescribed beneficial role for L1 activation in osteogenesis and enhanced understanding of the immediate molecular response to fracture within the bone marrow niche.
